# The causal relationship and potential mediators between plasma lipids and atopic dermatitis: a bidirectional two-sample, two-step mendelian randomization

**DOI:** 10.1186/s12944-024-02134-9

**Published:** 2024-06-22

**Authors:** Yuke Zhang, Bohan Zhang, Ru Wang, Xinghan Chen, Haitao Xiao, Xuewen Xu

**Affiliations:** Department of Plastic and Burn Surgery, West China Hospital, Sichuan University, No.37, Guoxue Alley, Chengdu, Sichuan 610041 China

**Keywords:** Plasma lipids, Atopic dermatitis, Inflammatory protein, Mendelian randomization

## Abstract

**Background:**

Observational studies have indicated that the plasma lipid profiles of patients with atopic dermatitis show significant differences compared to healthy individuals. However, the causal relationship between these differences remains unclear due to the inherent limitations of observational studies. Our objective was to explore the causal effects between 179 plasma lipid species and atopic dermatitis, and to investigate whether circulating inflammatory proteins serve as mediators in this causal pathway.

**Methods:**

We utilized public genome-wide association studies data to perform a bidirectional two-sample, two-step mendelian randomization study. The inverse variance-weighted method was adopted as the primary analysis technique. MR-Egger and the weighted median were used as supplementary analysis methods. MR-PRESSO, Cochran’s Q test, and MR-Egger intercept test were applied for sensitivity analyses to ensure the robustness of our findings.

**Results:**

The Mendelian randomization analysis revealed that levels of Phosphatidylcholine (PC) (18:1_20:4) (OR: 0.950, 95% CI: 0.929–0.972, *p* = 6.65 × 10^− 6^), Phosphatidylethanolamine (O-18:1_20:4) (OR: 0.938, 95% CI: 0.906–0.971, *p* = 2.79 × 10^− 4^), Triacylglycerol (TAG) (56:6) (OR: 0.937, 95% CI: 0.906–0.969, *p* = 1.48 × 10^− 4^) and TAG (56:8) (OR: 0.918, 95% CI: 0.876–0.961, *p* = 2.72 × 10^− 4^) were inversely correlated with the risk of atopic dermatitis. Conversely, PC (18:1_20:2) (OR: 1.053, 95% CI: 1.028–1.079, *p* = 2.11 × 10^− 5^) and PC (O-18:1_20:3) (OR: 1.086, 95% CI: 1.039–1.135, *p* = 2.47 × 10^− 4^) were positively correlated with the risk of atopic dermatitis. The results of the reverse directional Mendelian randomization analysis indicated that atopic dermatitis exerted no significant causal influence on 179 plasma lipid species. The level of circulating IL-18R1 was identified as a mediator for the increased risk of atopic dermatitis associated with higher levels of PC (18:1_20:2), accounting for a mediation proportion of 9.07%.

**Conclusion:**

Our research suggests that plasma lipids can affect circulating inflammatory proteins and may serve as one of the pathogenic factors for atopic dermatitis. Targeting plasma lipid levels as a treatment for atopic dermatitis presents a potentially novel approach.

**Supplementary Information:**

The online version contains supplementary material available at 10.1186/s12944-024-02134-9.

## Introduction

Atopic dermatitis (AD) is a prevalent chronic inflammatory skin disease, characterized by dry skin, itchiness, and erythema. It often begins in childhood and can persist into adulthood [1]. AD significantly impacts the quality of life of patients and imposes a severe socioeconomic burden [2]. Despite extensive reports have enriched our understanding of AD, its precise pathogenic mechanisms remain elusive. Multiple risk factors, such as skin barrier deficiencies, mutations of the filaggrin gene, lower mean temperatures, lower relative humidity, and abnormal infiltration of helper T cells, all contribute to the onset of the disease [3, 4].

Alterations in plasma lipid levels have been observed across various diseases. Specifically, levels of ceramide and other sphingolipids have emerged as crucial for both diagnosis and treatment in cardiometabolic diseases such as hypertension, atherosclerosis, and diabetes, as well as neurodegenerative disorders including Alzheimer’s and Parkinson’s diseases [5, 6]. Additionally, connections between plasma lipid disturbances and inflammatory disorders, including Rheumatoid arthritis, inflammatory bowel diseases, and psoriasis, have been identified [7–9]. While significant research has focused on the role of stratum corneum lipids in skin barrier function, only a limited number of studies have examined the link between circulating lipids and AD, leaving their causal relationship unclear [10–12]. Understanding the causal relationship between circulating lipids and AD could enhance our comprehension of pathogenesis of AD and identify new therapeutic targets.

Mendelian randomization (MR) offers a novel perspective in epidemiological studies by using genetic variants as instrumental variables (IVs) to determine causal relationships between exposures and outcomes [13]. MR parallels the principles of randomized controlled trials, as alleles are randomly distributed at birth [14]. Compared to traditional observational studies, MR overcomes their limitations, such as the inability to ascertain causal relationships between phenomena and the impact of potential confounders on the accuracy of results [15].

In this article, we aim to employ the latest genome-wide association studies (GWAS) data and the MR approach to assess the causality of 179 plasma lipid species on AD. Given the strong link between circulating inflammatory proteins and both plasma lipids and AD, we also investigate whether inflammatory proteins mediate the impact of plasma lipids on AD risk.

## Materials and methods

### Study design

**Fig. 1:**
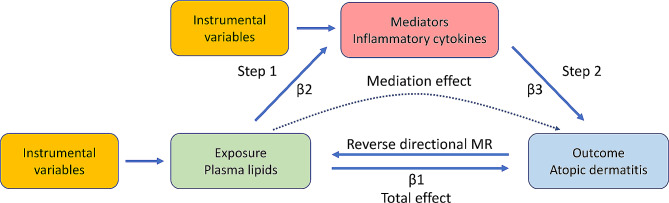
Overview of the study design. Mediation effect = β2 × β3. Mediation proportion = β2 × β3/β1

The flowchart of our study is depicted in Fig. 1. We initially utilized two-sample MR to examine the causal effects of 179 plasma lipid species on the risk of AD. Subsequently, to determine the presence of a bidirectional causal relationship between plasma lipids and AD, we performed reverse directional MR analysis, treating AD as the exposure and plasma lipids as the outcome. Following this, a two-step MR analysis was conducted to explore the potential mediation of inflammatory proteins in the relationship between plasma lipids and AD risk. Plasma lipids identified as having a causal effect on AD risk were further analyzed for their impact on 91 inflammatory proteins using two-sample MR. Finally, we evaluated the causal effects of inflammatory proteins, which are causally related to plasma lipids, on AD risk using two-sample MR. The method for calculating the mediation effect is determined by the formula: β2 × β3. Correspondingly, the proportion of mediation is calculated using the formula: β2 × β3/β1.

### Data sources

The GWAS data on plasma lipids were sourced from the study by Ottensmann et al., reporting GWAS findings on 179 lipid species across 13 lipid classes in 7,174 Finnish individuals from the GeneRISK cohort [16]. The GWAS data for circulating inflammatory proteins originated from a genome-wide protein quantitative trait locus study by Zhao et al., analyzing 91 inflammation-related proteins in 14,824 participants of European ancestry [17]. The GWAS data for AD was obtained from a GWAS meta-analysis that included 40 cohorts, totaling 448,060 AD cases and 3,321,586 controls of European ancestry [18]. All mentioned GWAS data are publicly available through the GWAS Catalog (https://www.ebi.ac.uk/gwas/home). Detailed information on data sources is provided in Table S1.

### Selection of IVs

In the MR studies, single-nucleotide polymorphisms (SNPs) served as IVs to represent the exposures. These IVs were required to fulfill three assumptions: (1) a strong correlation between IVs and the exposure; (2) IVs must be independent of any confounders that affect both the exposure and outcome; (3) IVs influencing the outcome exclusively through the exposure [19].

In our study, SNPs associated with AD at a genome-wide significance level (*p* < 5 × 10^− 8^) were selected. For inflammatory proteins and plasma lipids, a threshold of *p* < 5 × 10^− 6^ was adopted to ensure a sufficient pool of SNPs for the sensitivity analysis. Subsequently, SNPs with a minor allele frequency (MAF) > 0.01 were retained. SNPs were then filtered to remove linkage disequilibrium using data from the European samples of the 1000 Genomes Project (r^2^ = 0.001, clumping window = 10,000 kb). LDtrait (https://ldlink.nih.gov/?tab=ldtrait) was employed to check for associations between SNPs and potential confounding factors. Based on a previously published study, SNPs associated with obesity, allergy, smoking, and gut microbiota were excluded at a threshold of *p* < 5 × 10^− 8^ to mitigate the risk of pleiotropy [20, 21]. The SNPs excluded due to their association with potential confounding factors are presented in Table S2. The strength of the genetic instruments was evaluated using the F-statistic, with the calculation formula given as F = R^2^(*N* − 2)/(1 − R^2^). R^2^ represents the proportion of the phenotypic variance explained by each instrument. N denotes the sample size of the GWAS data [22]. The R^2^ is calculated using the formula R^2^ = (2 × EAF × (1 − EAF) × beta^2^)/[(2 × EAF × (1 − EAF) × beta^2^) + (2 × EAF × (1 − EAF) × N × se^2^)]. EAF is the effect allele frequency, beta is the estimated genetic effect on the exposures, se is the standard error of the genetic effect, and N represents the sample size of the GWAS data. SNPs with an F-statistic < 10 were discarded. The “TwoSampleMR” R package was used to align alleles between exposures and outcomes. The palindromic SNPs with a MAF > 0.42 were removed. The refined SNPs were utilized as IVs for subsequent MR analyses.

### Two-sample MR

The primary analytical method in our study was the Multiplicative random-effects inverse variance weighted (IVW) approach, supplemented by MR-Egger and weighted median methods. Sensitivity analyses, including Cochran’s Q test for heterogeneity among IVs, MR-Egger intercept test, and MR-pleiotropy residual sum and outlier (MR-PRESSO) for assessing horizontal pleiotropy, were conducted. MR-PRESSO was also used to identify and exclude outliers, with the MR analysis repeated until no outliers remained [23]. Leave-one-out analysis assessed the impact of individual SNPs on the overall causal inference.

### Statistical analysis

All analyses were executed using the TwoSampleMR, MRPRESSO, MendelianRandomization and RMediation packages in R software version 4.3.1. Significance was determined using the Bonferroni correction method (*p* < 0.05/n, where n represents the product of the number of tested exposures and outcomes). Results with *p* < 0.05 but not meeting the Bonferroni correction threshold were considered to indicate a suggestive association. Standard errors and confidence intervals (CIs) of mediation effects were calculated using the delta method [24].

## Results

### Genetic instruments

The SNPs utilized as IVs for exposures are detailed in Tables S3-5. As described in the Materials and Methods section, these SNPs underwent additional screening prior to the MR analysis. SNPs with an F-statistic < 10 were excluded to mitigate the risk of bias in MR effect estimates attributable to weak IVs. The palindromic SNPs with a MAF > 0.42 were removed. Following each MR analysis, MR-PRESSO was employed to identify outliers, and the analysis was repeated. This process was continued until MR-PRESSO did not detect any outliers. SNPs excluded by MR-PRESSO are listed in Table S6.

### Bidirectional MR analysis

#### The causal effects of plasma lipids on AD

**Fig. 2:**
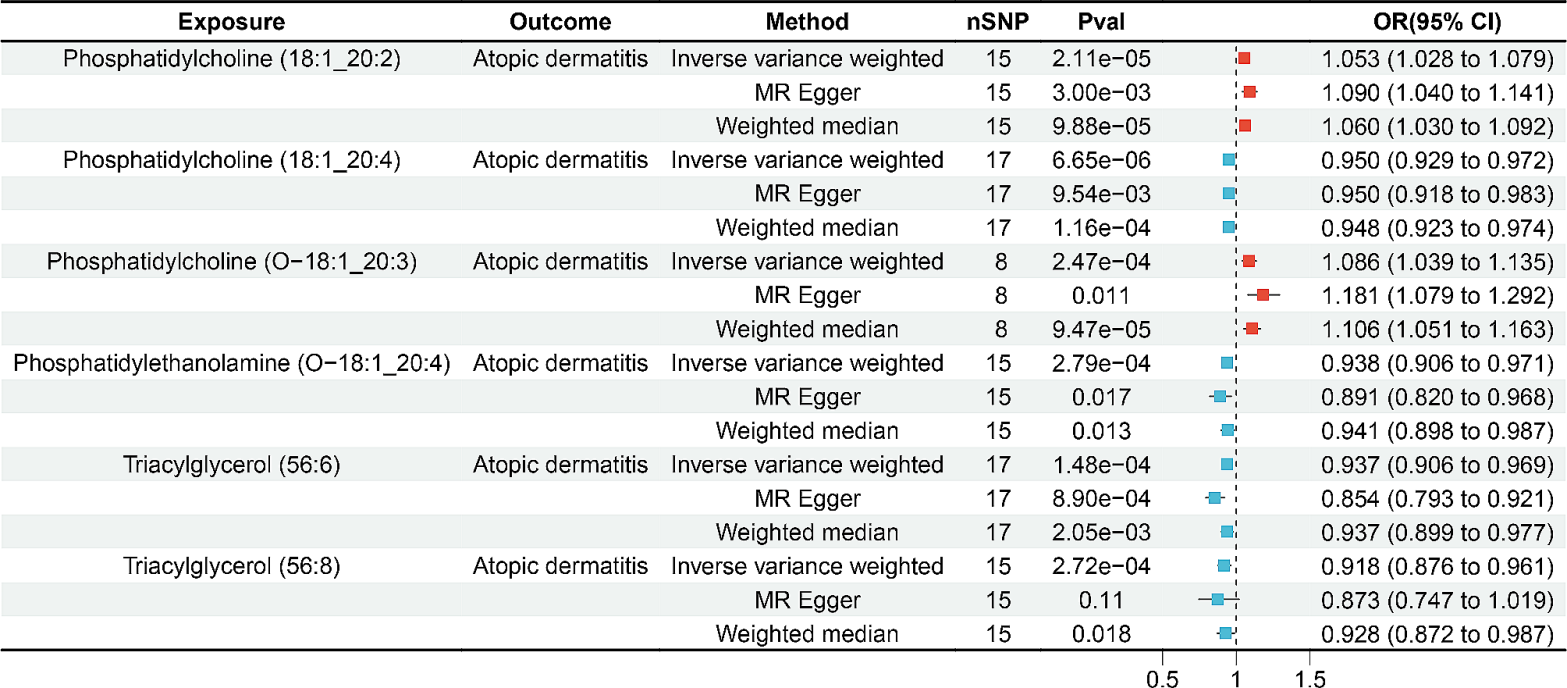
Two-sample MR results of the causal effects of plasma lipids on AD risk. nSNP, number of single-nucleotide polymorphisms; OR, odds ratio; CI, confidence interval

In this analysis, the Bonferroni correction method was applied to adjust p-values. Results with *p* < 2.79 × 10^− 4^ (0.05/179 exposures) were considered statistically significant. After applying the Bonferroni correction, the IVW method indicated that six plasma lipid species were significantly associated with the risk of AD. Phosphatidylcholine (PC) (18:1_20:4) (OR: 0.950, 95% CI: 0.929–0.972, *p* = 6.65 × 10^− 6^), Phosphatidylethanolamine (PE) (O-18:1_20:4) (OR: 0.938, 95% CI: 0.906–0.971, *p* = 2.79 × 10^− 4^), Triacylglycerol (TAG) (56:6) (OR: 0.937, 95% CI: 0.906–0.969, *p* = 1.48 × 10^− 4^) and TAG (56:8) (OR: 0.918, 95% CI: 0.876–0.961, *p* = 2.72 × 10^− 4^) reduced susceptibility to AD, while PC (18:1_20:2) (OR: 1.053, 95% CI: 1.028–1.079, *p* = 2.11 × 10^− 5^) and PC (O-18:1_20:3) (OR: 1.086, 95% CI: 1.039–1.135, *p* = 2.47 × 10^− 4^) increased AD risk (Fig. 2). Detailed results of the MR analysis, demonstrating causal effects of plasma lipids on atopic dermatitis with *p* < 0.05 using the IVW method, were presented in Table S7.

#### The causal effects of AD on plasma lipids

The reverse-direction MR analysis suggested potential causal effects of AD on four plasma lipid species. However, after the Bonferroni correction, all results were no longer statistically significant. Furthermore, there was no evidence of a potential bidirectional causal relationship between any plasma lipid species and AD (Table S8).

### Two-step MR Analysis

#### The causal effects of plasma lipids on circulating inflammatory proteins

**Fig. 3:**
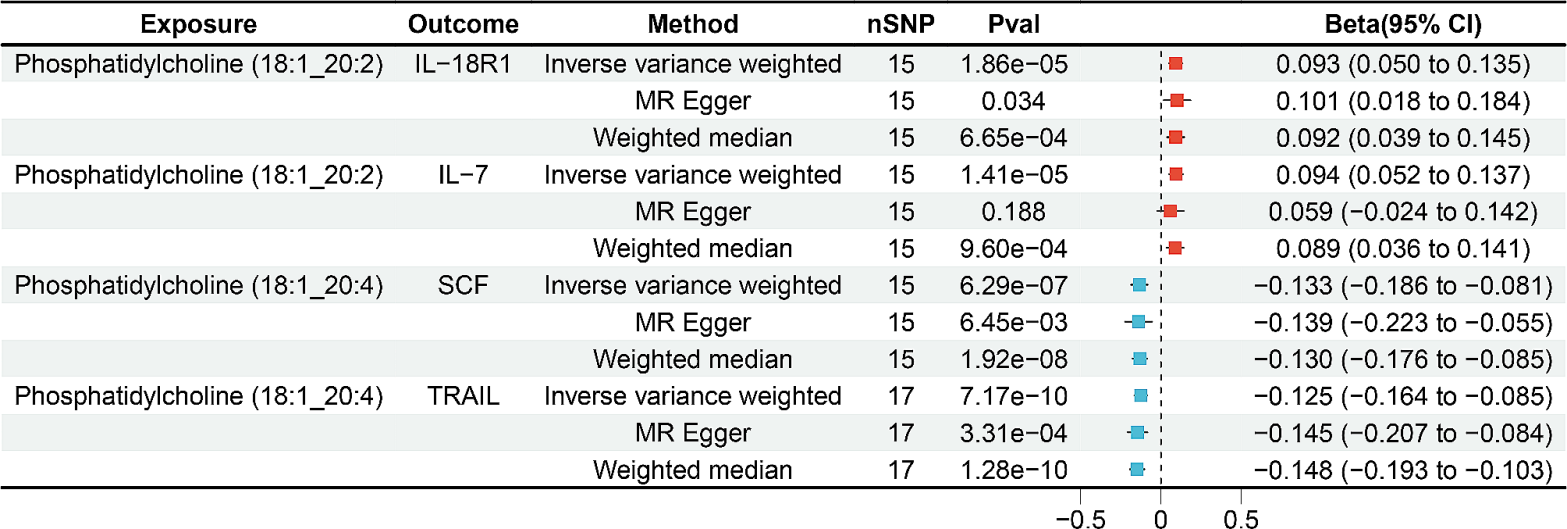
Two-sample MR results of the causal effects of plasma lipids on circulating inflammatory proteins. nSNP, number of single-nucleotide polymorphisms; Beta, estimated effect size of the IVs on the outcome; CI, confidence interval

Given the close association of inflammatory proteins with both plasma lipids and AD, we hypothesized that inflammatory proteins mediate the effect of plasma lipids on the risk of AD. From the results above, six plasma lipid species showed significant causal associations with AD risk. These six plasma lipids were selected and analyzed for their causal relationships with 91 inflammatory proteins. Using the IVW method as the primary analytical approach and after applying the Bonferroni correction (*p* < 9.16 × 10^− 5^ (0.05/(6 exposures × 91 outcomes)), we identified four significant associations. PC (18:1_20:4) was negatively associated with the levels of stem cell factor (SCF) (β: -0.133, 95% CI: -0.186 to -0.081, *p* = 6.29 × 10^− 7^) and TNF-related apoptosis-inducing ligand (TRAIL) (β: -0.125, 95% CI: -0.164 to -0.085, *p* = 7.17 × 10^− 10^). PC (18:1_20:2) showed positive associations with the levels of interleukin-18 receptor 1 (IL-18R1) (β: 0.093, 95% CI: 0.050 to 0.135, *p* = 1.86 × 10^− 5^) and Interleukin-7 (IL-7) (β: 0.094, 95% CI: 0.052 to 0.137, *p* = 1.41 × 10^− 5^) (Fig. 3). Detailed results of the MR analysis, indicating causal effects of plasma lipids on inflammatory proteins with *p* < 0.05 using the IVW method, were presented in Table S9.

#### The causal effects of circulating inflammatory proteins on AD

**Fig. 4:**
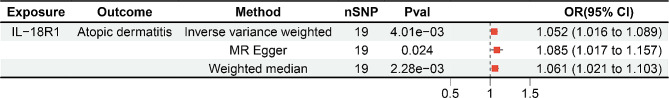
Two-sample MR results of the causal effects of circulating inflammatory proteins on AD risk. nSNP, number of single-nucleotide polymorphisms; OR, odds ratio; CI, confidence interval

In the previous analysis, four inflammatory proteins were significantly associated with plasma lipids. At this stage, we examined the relationship between these four inflammatory proteins and AD. The IVW analysis method revealed a significant association between IL-18R1 and the risk of AD (OR: 1.052, 95% CI: 1.016–1.089, *p* = 4.01 × 10^− 3^) (Fig. 4, Table S10). This result remained significant after applying the Bonferroni correction (*p* < 0.0125 (0.05/4 exposures)).

### Mediation proportion

**Fig. 5:**
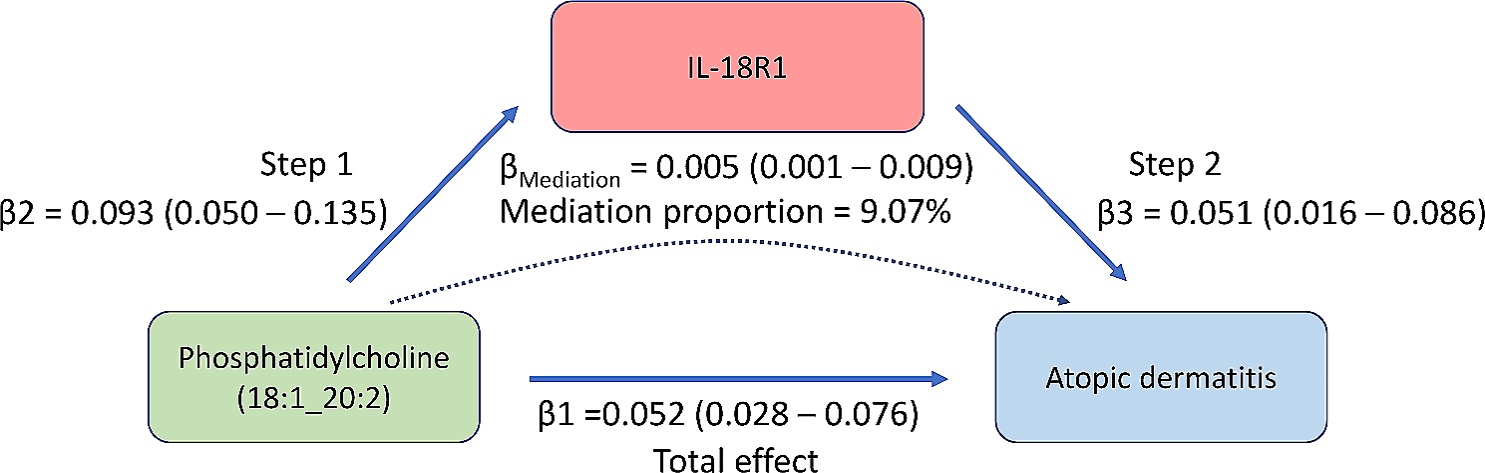
The mediation effect of IL-18R1 in the causal effect of PC (18:1_20:2) on AD risk

Through our analysis, we identified a single mediator, IL-18R1, which mediated the effect of PC (18:1_20:2) on AD risk. The mediation effect and proportion were calculated using the delta method. The results indicated that the mediation effect of IL-18R1 was 0.005 (95% CI: 0.001–0.009, *p* = 0.019), accounting for 9.07% of the total effect (Fig. 5).

### Sensitivity analyses

As described in the Materials and Methods section, we repeatedly used MR-PRESSO to eliminate outliers before arriving at the final results. For the final outcomes, Cochran’s Q test was utilized to assess heterogeneity. Among all results that remained significant associations after Bonferroni correction, no heterogeneity was detected. MR-Egger intercept test indicated potential horizontal pleiotropy in the results of TAG (56:6) on AD. However, another method for detecting horizontal pleiotropy, the MR-PRESSO global test, did not identify the presence of horizontal pleiotropy. No horizontal pleiotropy was found in other results from the MR-Egger intercept and MR-PRESSO tests (Tables S11-14). Fig S1-6 displayed the funnel plots and leave-one-out analyses for MR results that maintained significant causal associations after Bonferroni correction. The symmetry of some funnel plots suggested the presence of potential horizontal pleiotropy. However, due to the limited number of instrumental variables, it was difficult to assess the symmetry of the funnel plots. The results of the leave-one-out analysis indicated that the overall findings were not influenced by any single SNP.

## Discussion

In this study, we employed a bidirectional two-sample and two-step MR analysis to explore the causal relationship between 179 plasma lipid species and AD as well as potential mediators. Our findings indicate a negative correlation between the levels of PC (18:1_20:4), PE (O-18:1_20:4), TAG (56:6), and TAG (56:8) and the AD risk. Conversely, the levels of PC (18:1_20:2) and PC (O-18:1_20:3) were positively correlated with AD risk. Subsequently, we applied a two-step MR analysis to examine if circulating inflammatory proteins mediate the impact of plasma lipids on AD risk. The results of the first step revealed a causal connection between four inflammatory proteins and two plasma lipids associated with AD risk. The second step showed that only IL-18R1 among these four inflammatory proteins has a positive causal relationship with AD risk. Ultimately, we found that the increased risk of AD caused by PC (18:1_20:2) is partly mediated by IL-18R1.

PC is the most abundant phospholipid in all mammalian cell types and subcellular organelles [25]. Besides playing a crucial role in maintaining cellular membrane integrity, recent research has underscored the significant role of PC in the pathogenesis of various diseases, highlighting its potential as a therapeutic agent. Specifically, studies have indicated that PC could offer new therapeutic pathways for treating Inflammatory Bowel Disease by affecting gut barrier functions, modulating gut microbiota, and reducing inflammation [26]. Moreover, the metabolism of PC by gut flora has been implicated in the promotion of cardiovascular disease. This association is due to the metabolic transformation of dietary PC into proatherogenic metabolites like trimethylamine N-oxide, which has been linked to an increased risk of cardiovascular diseases [27]. Beyond the role of PC in gut metabolism, the association between changes in plasma PC levels and diseases has garnered increasing attention, such as in Alzheimer’s disease, rheumatoid arthritis, depression, and cancer [28–31].

In the case of AD, a recent observational study suggested that compared to healthy individuals, patients with AD exhibit a dysregulation of various lipid levels in their serum. However, due to the limitations of observational studies, it is challenging to establish a causal relationship between circulating lipids and AD. Our use of bidirectional MR analysis identified six plasma lipid species significantly impacting AD risk, with no evidence suggesting that AD affects plasma lipid levels, implying that plasma lipids might be one of the pathogenic factors of AD. Notably, PC (18:1_20:4), PC (18:1_20:2) and PC (O-18:1_20:3), all belonging to the PC class, have opposite effects on AD risk. Unlike the bidirectional effects of PCs on AD risk, the results of our Mendelian randomization analysis suggest that two types of TAGs are associated with reduced susceptibility to AD. Normal epidermal triacylglycerol metabolism contributes to the synthesis of ω-(O)-acylceramides, which are essential for maintaining proper skin permeability barrier function [32]. However, previous studies have indicated that children with AD have significantly higher levels of serum TAGs compared to healthy children, and higher TAG levels are associated with more severe AD [33, 34]. This is inconsistent with some of our findings. This discrepancy may be due to the fact that the GWAS data for plasma lipids used in our study was derived from adults aged 45–66, leading to differences in the study populations. It is also possible that some types of TAGs are associated with the development of AD, but these were not covered in the data we used. Previous articles have pointed out that individual lipid species are more accurate than traditional lipid markers in predicting cardiovascular disease risk. This might also apply to AD [35].

The relationship between plasma inflammatory proteins and AD has been extensively reported. We utilized MR analysis to investigate whether plasma inflammatory proteins mediate the effect of plasma lipids on AD risk. The results showed that the levels of TRAIL, IL-18R1, IL-7, and SCF are associated with the levels of two plasma lipids impacting AD risk. Among these, only IL-18R1 was positively correlated with AD risk. In fact, a MR study published in 2021 already mentioned the positive correlation between IL-18R1 and AD [36, 37]. We further validated this result using a larger sample size and the latest GWAS data. IL-18R is a critical component of the IL-18 signaling pathway. IL-18R is comprised of two subunits, IL-18R1 and IL-18RAP. IL-18R1 is responsible for the binding to IL-18, while IL-18RAP contributes to signal transduction, enabling the activation of downstream signaling pathways, such as NF-κB, MAPK, JNK, and AKT. Moreover, IL-18R1 also participates in the regulation of immune cell phenotypes [38]. Research by Beyer et al. has shown that histamine and IFN-γ can synergistically upregulate the expression of IL-18R1 in eosinophils of AD patients. This promotive effect on IL-18R1 expression could lead to an increase in the expression of the eosinophil granule proteins [39]. Although in our study, only IL-18R1 was found to mediate the effect of plasma lipids on AD risk, part of our results indicates a significant correlation between plasma lipids and circulating inflammatory protein levels. This suggests that plasma proteins may mediate changes in plasma inflammatory protein levels, affecting the risk of developing various diseases.

Although we have elucidated the causal relationship between plasma lipids and AD risk through MR analysis, our study has certain limitations. Firstly, the GWAS data used in our study are all from European ancestry, hence, whether our results are applicable to other populations remains to be validated. Moreover, while IL-18R1 has been identified as a mediator, we were unable to elucidate the specific mechanisms, which necessitates further experimental validation. Additionally, plasma lipids and inflammatory proteins may be associated with multiple diseases, yet we only explored their relationship with AD.

## Conclusion

Our study demonstrated a causal relationship between plasma lipids and AD risk through MR analysis, with IL-18R1 emerging as a key mediator. These findings not only suggest that plasma lipids may be one of the pathological factors in AD, but also imply that plasma lipids hold promise as therapeutic targets.

### Electronic supplementary material

Below is the link to the electronic supplementary material.

Supplementary Material 1

Supplementary Material 2

Supplementary Material 3

Supplementary Material 4

Supplementary Material 5

Supplementary Material 6

Supplementary Material 7

## Data Availability

The datasets supporting the conclusions of this article are available in the GWAS Catalog (https://www.ebi.ac.uk/gwas/home).

## Abbreviations

AD: Atopic dermatitis
MR: Mendelian randomization
IVs: Instrumental variables
GWAS: genome-wide association studies
SNPs: Single-nucleotide polymorphisms
IVW: Inverse variance weighted
MR-PRESSO: MR-pleiotropy residual sum and outlier
CIs: Confidence intervals
PE: Phosphatidylethanolamine
PC: Phosphatidylcholine
TAG: Triacylglycerol
SCF: Stem cell factor
TRAIL: TNF-related apoptosis-inducing ligand
IL-18R1: Interleukin-18 receptor 1
IL-7: Interleukin-7
