# Supplementary figures and images for: The causal relationship and potential mediators between plasma lipids and atopic dermatitis: a bidirectional two-sample, two-step mendelian randomization
Source: Lipids Health Dis. 2024 Jun 22;23:191. doi: 10.1186/s12944-024-02134-9 (PMC11193249; doi:10.1186/s12944-024-02134-9)

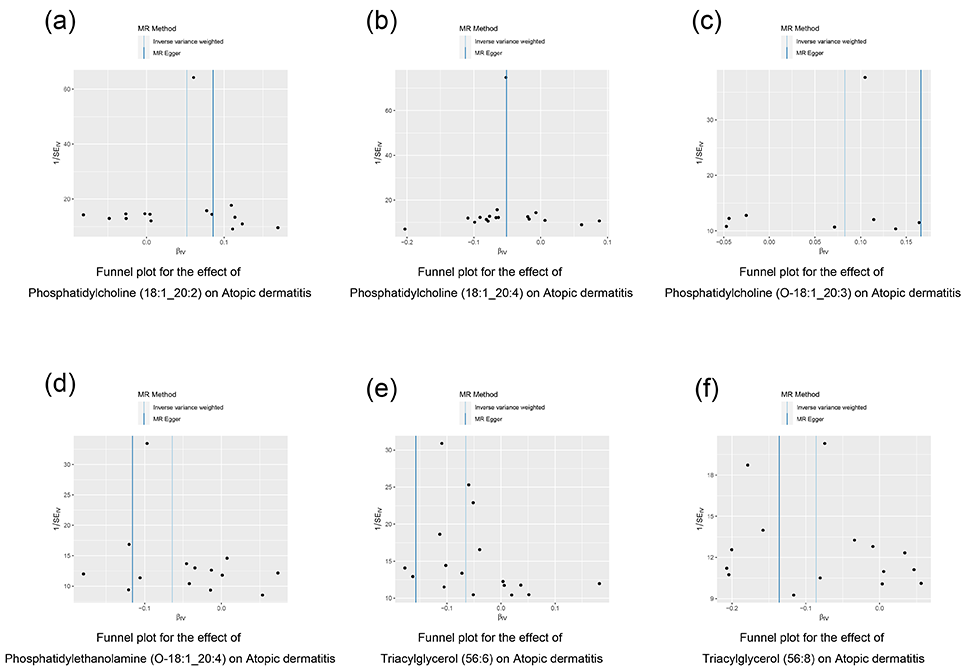

Supplement: Supplementary file 2 — Supplementary Material 2 [file 12944_2024_2134_MOESM2_ESM.tif]

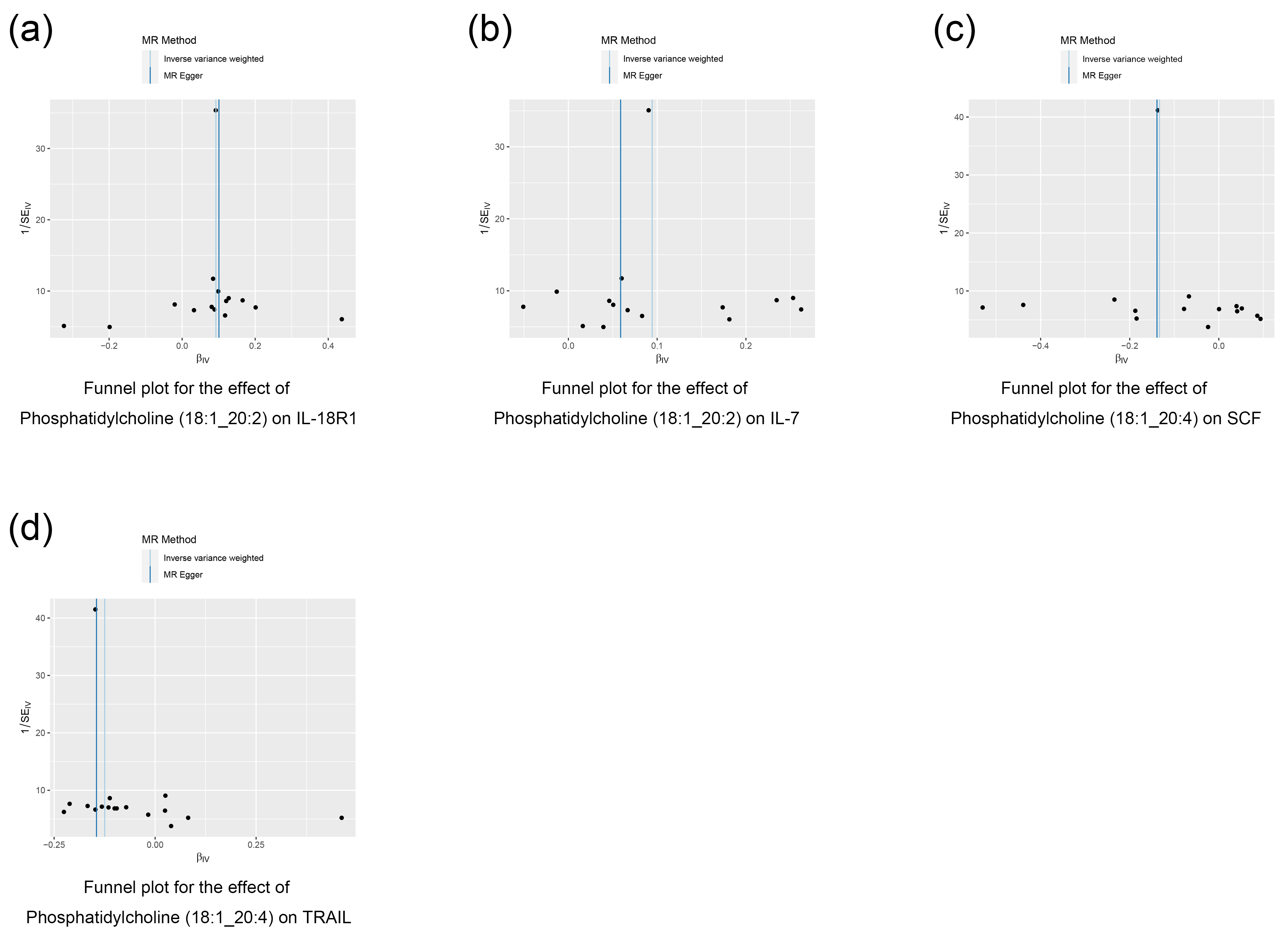

Supplement: Supplementary file 3 — Supplementary Material 3 [file 12944_2024_2134_MOESM3_ESM.tif]

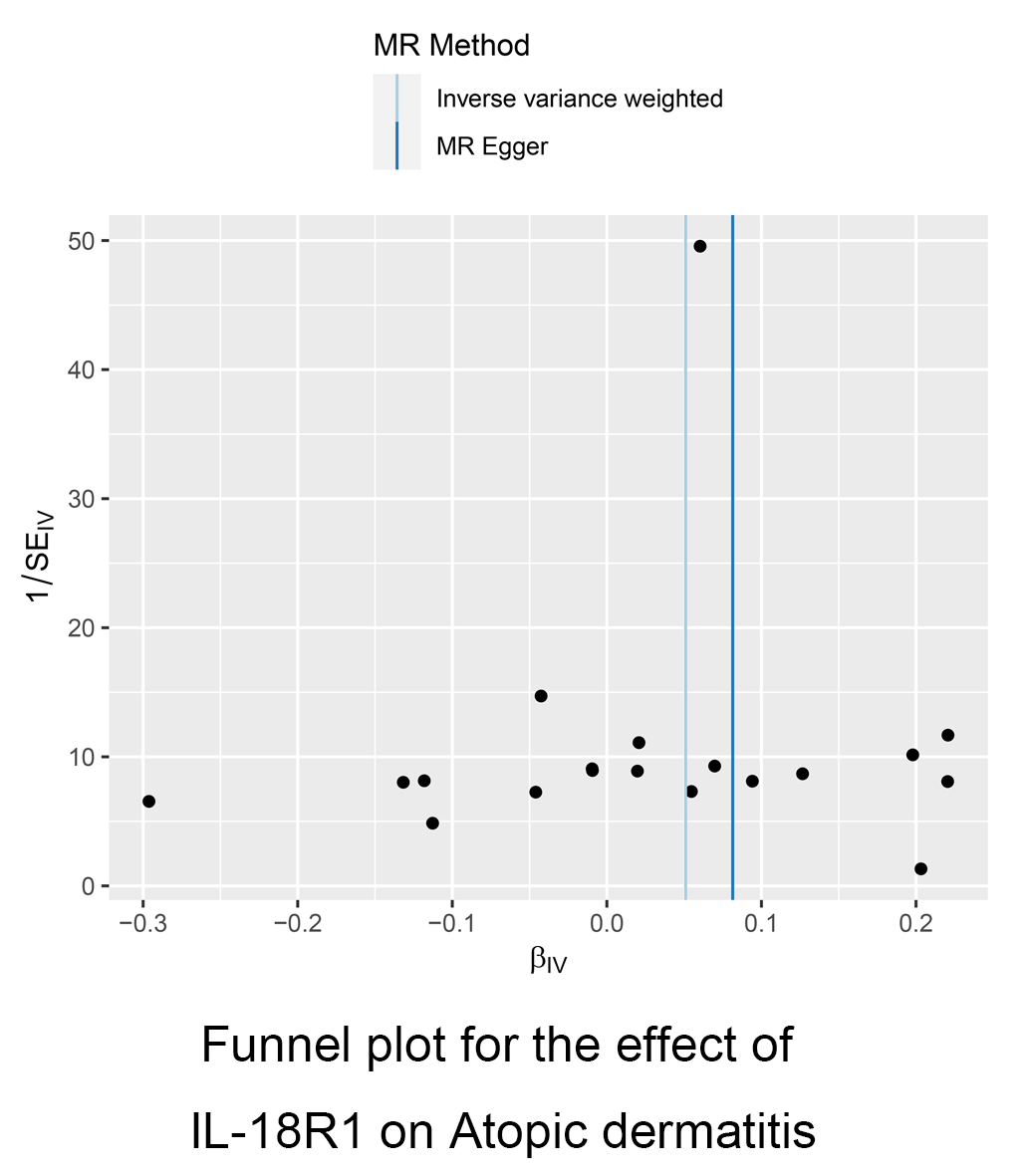

Supplement: Supplementary file 4 — Supplementary Material 4 [file 12944_2024_2134_MOESM4_ESM.tif]

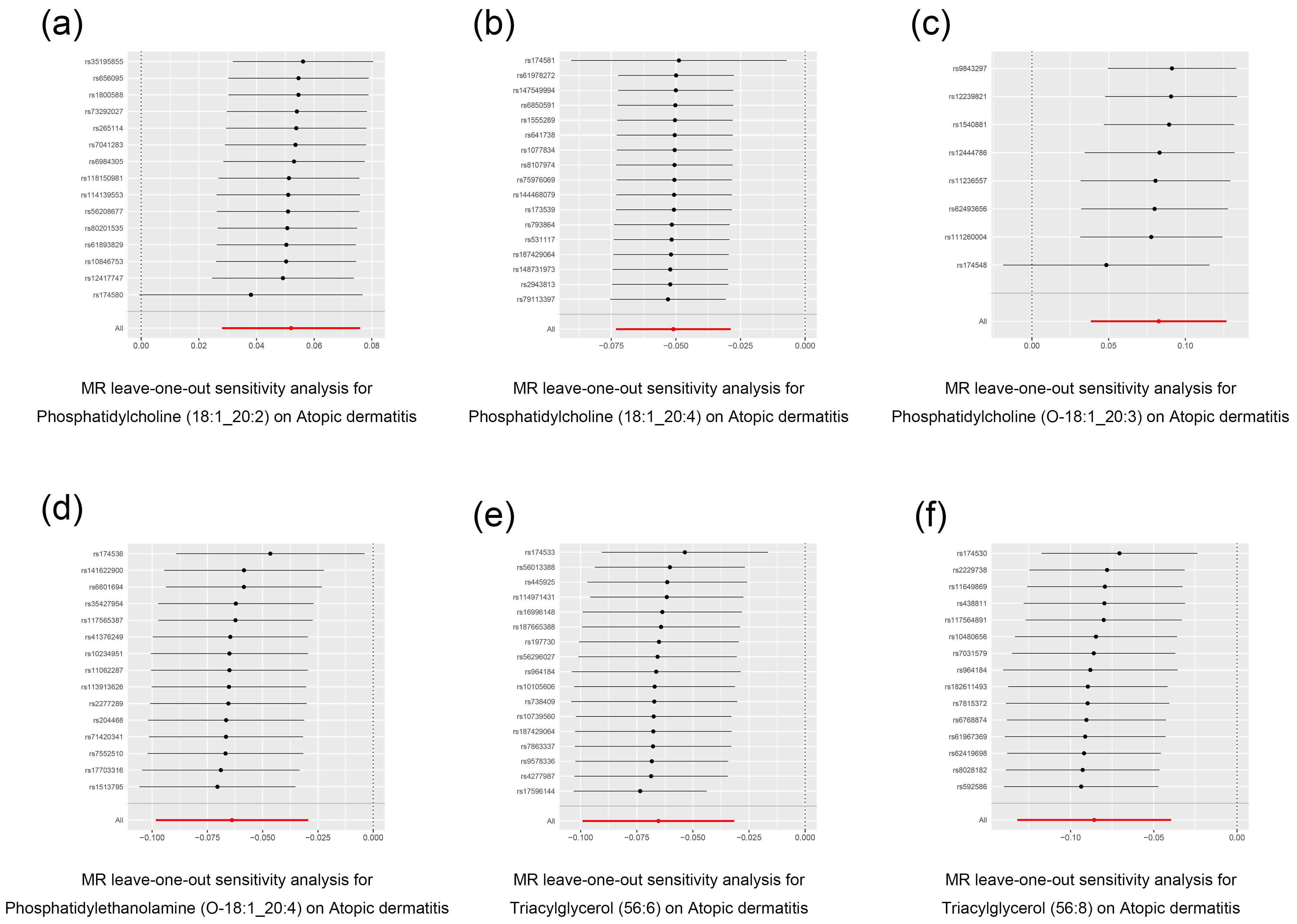

Supplement: Supplementary file 5 — Supplementary Material 5 [file 12944_2024_2134_MOESM5_ESM.tif]

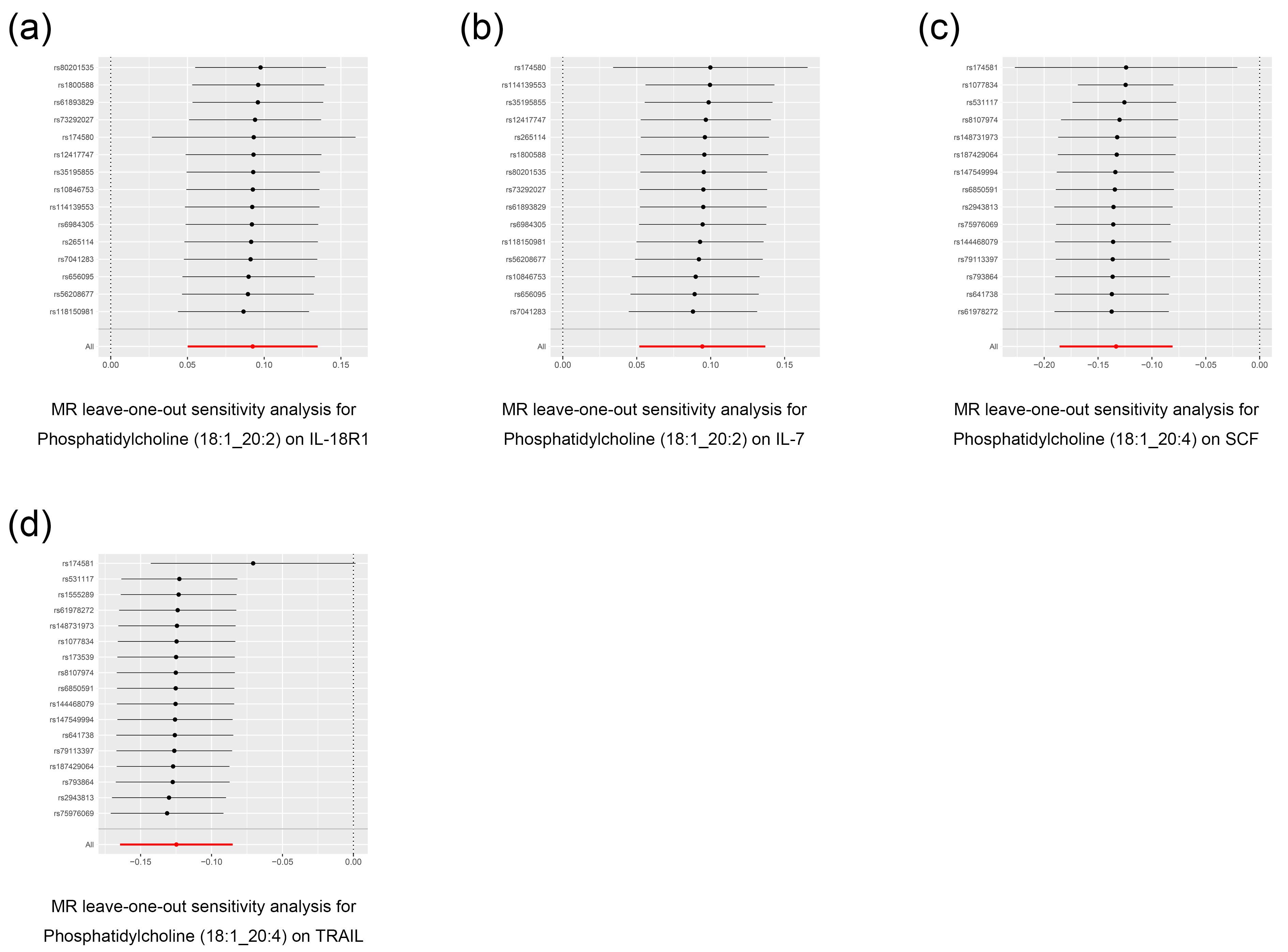

Supplement: Supplementary file 6 — Supplementary Material 6 [file 12944_2024_2134_MOESM6_ESM.tif]

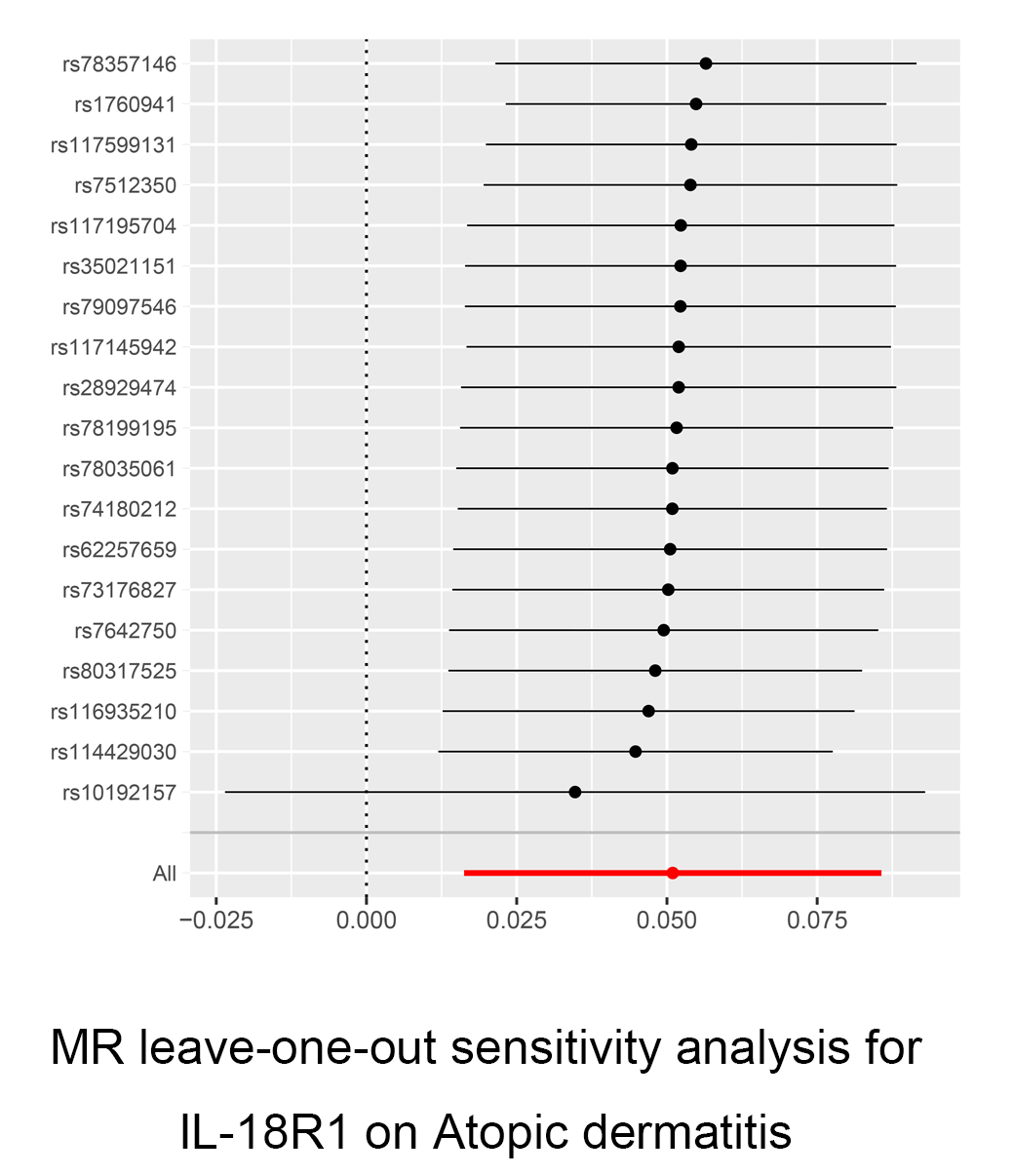

Supplement: Supplementary file 7 — Supplementary Material 7 [file 12944_2024_2134_MOESM7_ESM.tif]
